# Severe hyponatremia as the first sign of late-onset Schmidt’s syndrome: A case report

**DOI:** 10.22088/cjim.12.0.392

**Published:** 2021

**Authors:** Oskar Wojciech Wiśniewski, Paulina Matuszak, Agnieszka Kasprzak, Katarzyna Łącka

**Affiliations:** 1 Poznan University of Medical Sciences, Poznan, Poland; 2 Department of Internal Diseases and Diabetics, Heliodor Swiecicki Clinical Hospital, Poznan, Poland; 3 Department of Endocrinology, Metabolism and Internal Medicine, Poznan University of Medical Sciences, Poznan, Poland

**Keywords:** Addison’s disease, Autoimmune thyroiditis, Hyponatremia, Schmidt’s syndrome

## Abstract

**Background:**

Schmidt’s syndrome (SS) is a rare endocrine disorder (14-20:1000000), which consists of autoimmune thyroiditis (AIT) and autoimmune Addison’s disease (aAD), and usually occurs in young adults. Here, we report a unique case of late-onset SS manifesting initially with isolated severe hyponatremia and present the hazardous outcomes of preliminary misdiagnosis.

**Case Presentation:**

A 78-year-old female presented to the emergency department with a two-day history of diarrhea, emesis and disturbances in consciousness. She also reported general fatigue and increasing weakness in the last month. Urgent laboratory findings revealed isolated severe hyponatremia (serum sodium=108 mmol/l) and initial treatment with active sodium infusions was started, although with no improvement in the patient's neurological status after 5 days (serum sodium=127 mmol/l). Meanwhile, the patient developed recurring episodes of hypoglycemia and symptoms portending adrenal crisis (blood pressure=105/58 mmHg, heart rate=96 bpm, severe whole-body muscle pain, two loose stools), which required immediate i.e. hydrocortisone treatment. Reduced blood cortisol, elevated adrenocorticotropic hormone (ACTH) and atrophic morphology of the adrenal glands in computed tomography imaging contributed to the final diagnosis of aAD and SS consequently, since the patient had a past medical history of AIT.

**Conclusion:**

Isolated severe hyponatremia should not be underestimated as the first sign of aAD. Appropriate cause-specific treatment is crucial in managing hyponatremia.

Schmidt’s syndrome (SS) is a subtype of autoimmune polyglandular syndrome type 2 (APS-2), a rare endocrine disorder characterized by the prevalence of 14-20:1000000 (1). It consists of autoimmune thyroiditis (AIT) and Addison’s disease (AD) of autoimmune origin, and usually occurs in women aged 21-40 (1, 2). The aim of our paper is to introduce the unique case report demonstrating the abrupt onset of SS in the middle-old population (age: 75-84) accompanied with severe hyponatremia and highlight the difficulties with appropriate causative diagnosis regarding severe hyponatremia as the only initial sign of SS and grievous consequences of misdiagnosis. 

## Case presentation

A 78-year-old female presented to the emergency department with a two-day history of diarrhea, emesis and severe neurological symptoms, such as confusion, disorientation and somnolence. She also reported general fatigue and increasing weakness in the last month. The initial physical examination revealed a dry tongue and oral cavity, as well as moistureless skin. 

Blood pressure (BP) was 105/60 mm Hg and heart rate (HR) 71 bpm. Initial laboratory results revealed a serum sodium concentration of 108 mmol/l, indicative of severe hyponatremia, and no aberrations in blood glucose (92 mg/dl) and potassium (3.85 mmol/l). No signs of heart failure, liver impairment and renal dysfunction were observed on clinical, imaging or laboratory investigations.

Past medical history included hypothyroidism in the course of AIT, which was diagnosed ten years ago and might be associated with the presence of hyponatremia, dry skin, general malaise and weakness. Nevertheless, the patient was euthyroid on admission (thyroid-stimulating hormone=2.16 µU/ml; free triiodothyronine=4.03 pmol/l; free thyroxine=17.13 pmol/l), supplementing 25 µg of levothyroxine daily, which excluded the role of thyroid disease in the pathogenesis of the abovementioned signs and symptoms. 

In addition, the patient reported no suspicion of any other autoimmune disease in the medical history, such as autoimmune AD (aAD), type 1 diabetes, premature ovarian insufficiency, vitamin B12 deficiency, vitiligo, alopecia or autoimmune hepatitis, and denied sudden drop in blood pressure or hypoglycemic attack. In-depth anamnesis disclosed sedentary lifestyle, daily intake of 2-2.5 L of fluids, no specific dietary discipline as well as no alcohol abuse or smoking. Moreover, the patient suffered from arterial hypertension treated with metoprolol in monotherapy.

As the initial diagnosis was severe hypovolemic hyponatremia with emesis and diarrhea considered as the most likely cause, the patient was referred to the Internal Medicine ward, where active treatment with 0.9% NaCl enriched with 10% NaCl was introduced. However, after 5 days of such a therapy, the patient’s neurological status had not improved, although the serum sodium level had risen gradually from 108 mmol/l to 127 mmol/l. Due to persisting intense neurological symptoms, computed tomography (CT) of the head was performed and showed a meningioma (20x12 mm) in the left occipital region. 

However, it transpired that the meningioma had been found 3 years ago and the patient was under permanent neurological care (watchful waiting strategy; the last consultation a month before admission). During the first 24 hours of hospitalization, the patient developed recurring episodes of hypoglycemia (blood glucose=45-49-54 mg/dl), which led to reassessment of the patient’s condition. A detailed examination exhibited slight skin hyperpigmentation. At that time, the first suspicion of adrenal insufficiency emerged and pituitary-adrenal axis hormones measurements were performed (table 1), disclosing decreased blood cortisol and augmented adrenocorticotropic hormone (ACTH).

**Table 1:** Alterations in pituitary-adrenal axis hormone concentrations

	***Day of therapy***	***Morning serum cortisol nmol/l*** ***NR (110-692)***	***Morning serum ACTHpg/ml*** ***NR (7,20-63,30)***
Before replacement therapy	-5^th^ day	61	1066
After replacement therapy	1^st^ day	>1750	97,39
	16^th^ day	24	27,44

ACTH: adrenocorticotropic hormone; NR: normal range

**Figure 1:**
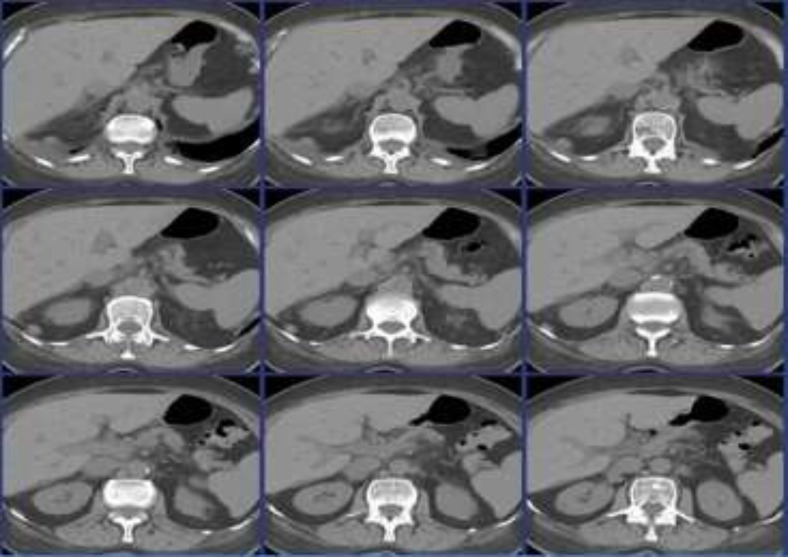
Atrophic morphology of the adrenal glands in contrast CT imaging

Meanwhile, while waiting for the hormonal measurement results, the patient developed symptoms portending adrenal crisis. The patient reported whole-body muscle pain, which was not responding well to regular treatment and was characterized by elevated creatine kinase (CK; 1494 U/l), creatine kinase-MB (CK-MB; 5.53 ng/ml) and lactate dehydrogenase (LDH; 259 U/l), indicative of rhabdomyolysis. In addition, the patient excreted two loose stools. Cardiovascular measurements revealed mild hypotension (BP=105/58 mm Hg) and sinus rhythm on the very edge of tachycardia (HR=96 bpm). Considering the set of hypocortisolism symptoms, immediate treatment with *i.v.* hydrocortisone was started. The next day, the patient reported a substantial improvement in pain management and a better state of being. Furthermore, serum sodium concentration exceeded 135 mmol/l and neurological ailments resolved. Contrast CT imaging unveiled atrophic morphology of the adrenal glands (figure 1), which led to the diagnosis of aAD.

Elevated anti-thyroid antibodies (anti-thyroid peroxidase antibodies=406 IU/ml; thyroglobulin antibodies=105 IU/ml) and hypoechogenic thyroid in the ultrasounds confirmed the diagnosis of AIT and the final diagnosis of SS was made. Glucocorticoid replacement therapy with 20 mg of oral hydrocortisone in the morning and 10 mg in the afternoon was implemented. Assessment of the therapy applied resulted in addition of 25 µg/d of fludrocortisone to the regimen (table 1). On the day of discharge and in a follow-up after two weeks, the patient reported no complaints and no abnormalities on examination or in laboratory results were found.

## Discussion

The patient presented to the hospital with a set of symptoms which cannot be clearly attributed to AD. Severe, acute hyponatremia could itself produce neurological disorders (3). In addition, emesis and diarrhea could be recognized as one of the most frequent causes of hypovolemic hyponatremia, misleading from other potential pathophysiological backgrounds resulting in hypovolemic hyponatremia, such as AD. 

Since the patient suffered from hyponatremia with reduced extracellular fluid (ECF), other causes resulting in euvolemic (e.g. syndrome of inappropriate antidiuretic hormone secretion, primary polydipsia), or hypervolemic hyponatremia (e.g. heart failure, cirrhosis, nephrotic syndrome, acute kidney injury, chronic kidney disease) should not be taken into account in the diagnostic process (4). In addition, a group of hypovolemic hyponatremia etiologies (e.g. third-spacing, diuretic excess and exercise-associated hyponatremia) were excluded on the basis of initial examination, anamnesis and medical history in the diagnostic process. Furthermore, severe hyponatremia without hyperkalemia as the first manifestation of AD is described in only a few cases (5-7), hence the initial treatment was focused on managing symptomatic hyponatremia itself. Finally, only the emergency administration of 100 mg *i.e.* hydrocortisone alleviated the patient’s neurological symptoms and aligned serum sodium concentration. Therefore, misconceiving the exact cause of hyponatremia may delay appropriate cause-specific treatment and leads to grievous consequences, or at least partial ineffectiveness of the therapy applied.

Clinical presentations of the mentioned cases (5-7) have a lot in common with our patient, since each of them reported general fatigue, emesis, hyperpigmentation and low blood pressure values. These similarities could be observed despite different patients’ characteristics, since the first case report described 32-year-old pregnant woman (5), the second referred to 10-year-old girl (6) and the third one regarded 44-year-old nonpregnant woman (7). However, unlike the mentioned cases, our patient did not report weight loss prior to hospitalization (5-7), nor had a history of premature ovarian insufficiency or severe arthralgia and myopathy before admission (7). In addition, rapid improvement in patients’ condition and management of hyponatremia were also observed in two of the cases just after hydrocortisone administration (5-6). Interestingly, while analyzing the relevant literature, we found that elevated levels of muscle damage markers (CK, CK-MB) at the time of AD diagnosis are rare and almost always coexist with severe hyponatremia (7-9), as in our case. The patient’s sequence of autoimmune disorders leading to APS-2 diagnosis is extraordinary, since hypothyroidism usually follows or coincides with the diagnosis of aAD (1). As long as 30-52% of patients suffering from APS-2 are affected with type 1 diabetes, our patient’s health check-ups should essentially include blood glucose measurements. Only 3 cases of severe hyponatremia accompanying the nonconcurrent diagnosis of SS in adrenal crisis or pre-adrenal crisis conditions have been reported so far in the PubMed database (table 2) (5, 10, 11). The same 3 cases also disclose severe hyponatremia without hyperkalemia. 

Nevertheless, our paper is not only the fourth such case, but primarily the first published case report of severe hyponatremia accompanying the diagnosis of SS in the elderly population (≥65 years). Late onset of SS coexisting with water-electrolyte disorders as a common issue of geriatric population should not be underestimated in the context of an ageing society.

**Table 2:** Overview of the literature

**Case report**	**Age and sex**	**serum [Na** ^+^ **]** ***mmol/l***	**serum ** **[K** ^+^ **]** ***mmol/l***	**Order of diagnosis** **(aAD and AIT)**	**Adrenal crisis or pre-adrenal ** **crisis conditions**
(5) Lewandowski K et al.	32 F	112	4.3	AIT before aAD	YES
(10) Tsang CC et al.	42 F	124	“normal”	AIT 3 years before aAD	YES
(11) Blevins CH et al.	52 F	120	4.5	AIT before aAD	YES

aAD: autoimmune Addison’s disease; AIT: autoimmune thyroiditis; F: female; [K^+^]: potassium concentration; [Na^+^]: sodium concentration

Taken together, potential coexistence of aAD should be suspected in each case of severe hyponatremia in patients previously diagnosed with AIT, independently of the absence of hyperkalemia and age. Thus, measurements of blood cortisol should always be made, if severe hyponatremia occurs in a patient with medical history of AIT. In addition, this patient should be carefully examined in search of hyperpigmentation, including oral cavity inspection. The presence of emesis, fatigue and low blood pressure increases the possibility of the adrenal origin of severe hyponatremia in AIT patients. The strengths of this case report include accurate imaging and the long period of observation in the inpatient’s clinic. However, there are also a few limitations, such as the lack of dehydroepiandrosterone sulfate and aAD-specific autoantibodies testing due to the hospital’s laboratory restrictions. Nevertheless, the results of the contrast CT imaging provided enough evidence to make the clinical diagnosis of aAD (12-14). 

In conclusion, severe hyponatremia may be the first sign of SS, occurring even in the middle-old population, and requires both symptomatic and causative treatment.
